# Emerging Molecular and Computational Biomarkers in Urothelial Carcinoma: Innovations in Diagnosis, Prognosis, and Therapeutic Response Prediction

**DOI:** 10.3390/jpm16010025

**Published:** 2026-01-05

**Authors:** Fernando Alberca-del Arco, Rocío Santos-Perez de la Blanca, Elisa Maria Matas-Rico, Bernardo Herrera-Imbroda, Félix Guerrero-Ramos

**Affiliations:** 1 Department of Urology, Hospital Universitario Virgen de la Victoria (HUVV), 29010 Malaga, Spain; fernando.alberca@ibima.eu (F.A.-d.A.); rocio.santosperez.sspa@juntadeandalucia.es (R.S.-P.d.l.B.); bernardo.herrera.imbroda.sspa@juntadeandalucia.es (B.H.-I.); 2 Instituto de Investigación Biomédica de Málaga y Plataforma en Nanomedicina (IBIMA Plataforma BIONAND), 29590 Malaga, Spain; elisa.matas@ibima.eu; 3 Genitourinary Alliance for Research and Development (GUARD Consortium); 4 Department of Cell Biology, Genetics and Physiology, Universidad de Málaga (UMA), 29071 Malaga, Spain; 5 Department of Surgical Specialties, Biochemistry and Immunology, Universidad de Málaga (UMA), 29071 Malaga, Spain; 6 Department of Urology, Hospital Universitario 12 de Octubre, Avda. Córdoba s/n, 28041 Madrid, Spain

**Keywords:** bladder cancer, urothelial carcinoma, biomarkers, multi-omics, artificial machine learning, immune checkpoint inhibitors, liquid biopsy, tumor microenvironment

## Abstract

Bladder cancer (BC) represents a major global health issue with high recurrence and significant mortality rates in cases of advanced disease. Currently, the development of molecular profiling, liquid biopsy technologies, and artificial intelligence (AI) software has resulted in unprecedented opportunities to improve diagnosis, prognostic assessment, and treatment selection. Recent multicenter studies have identified emerging metabolomic, proteomic, and genomic biomarkers with high sensitivity and specificity that may help replace or complement invasive approaches. AI-driven models that combine multi-omics datasets with radiomics and clinical parameters have demonstrated improved accuracy for predicting both therapeutic response and long-term outcomes, compared to standard approaches for risk stratification. Additionally, the incremental clinical usefulness of liquid biopsy platforms has been demonstrated for the monitoring of non-muscle-invasive bladder cancer and minimal disease detection. As these innovations converge, they herald the advent of a new era of personalized management of urothelial carcinoma; however, broad-based clinical implementation will require large-scale validation, standardization, regulatory harmonization, and economic analyses. Background: Bladder cancer continues to be a global health problem, particularly in the advanced disease setting where treatment options are limited, and mortality remains high. The exciting advances in precision medicine, including breakthrough molecular profiling techniques, liquid biopsy, and opportunities to apply AI to interpret these molecular data, hold unprecedented promise in improving the accuracy of diagnosis, prognostic stratification, and therapeutic decision-making.

## 1. Introduction

Bladder cancer is the ninth most common malignancy. Approximately 90% of all bladder cancers are urothelial carcinoma (UC). About 75% of patients at diagnosis have non-muscle invasive bladder cancer (NMIBC), whose clinical behavior can range from indolent to highly aggressive and life-threatening. Clinicopathological parameters help us to stratify risk into low-, intermediate-, and high-risk groups [1]. The rates of progression at 5 years widely vary according to the risk group, from 0.93% in low-risk NMIBC to 40% in very high-risk patients.

The emergence of personalized medicine is revolutionizing the management of UC, a disease characterized by high biological heterogeneity. At the same time, the development of techniques such as tumor molecular profiling and immunological assessment, together with minimally invasive diagnostic tools, is translating into a more individualized and precision approach for the management of this malignancy.

The stepwise accumulation of genetic alterations in key pathways of cell cycle regulation, apoptosis, signal transduction, and immune evasion underlies tumor progression along this spectrum. Among non-invasive diseases, exophytic papillary (Ta-T1) tumors and carcinoma in situ (CIS) are two groups with specific molecular characteristics. This is seen in the case of low-grade Ta tumors that often carry activating mutations in fibroblast growth factor receptor 3 (FGFR3) and Harvey rat sarcoma viral oncogene homolog (HRAS), leading to activation of receptor tyrosine kinase–RAS–MAPK signaling [2,3,4], whereas a high-grade Ta tumor more frequently involves homozygous deletion of p16^INK4a and shares a reduced frequency with the FGFR3 mutation [5]. CIS, on the other hand, is considerably more aggressive and associated with alterations of tumor protein 53 (TP53) and retinoblastoma (RB) pathways [6], alterations that also characterize invasive tumors (T2–T4), which acquire further molecular events affecting vascular endothelial growth factors (VEGF), cadherins, matrix metalloproteinases (MMPs), resulting in extracellular matrix remodeling, angiogenesis, and metastatic spread [7].

In this review, we aim to provide an update on the role of emerging molecular and computational biomarkers in the diagnosis, prognosis, and treatment response prediction of UC.

## 2. Materials and Methods

This narrative review was conducted to provide a comprehensive and up-to-date synthesis of the role of emerging molecular and computational biomarkers in the diagnosis, prognosis, and treatment response prediction of UC. A systematic search of the literature was performed using the PubMed/MEDLINE, Embase, Cochrane Library, and Web of Knowledge databases to identify relevant publications between January 2012 and August 2025. The search strategy combined medical subject headings (MeSH) and free-text terms including “muscle-invasive bladder cancer,” “urothelial carcinoma,” “molecular biomarkers,” “taxonomic subgroups,” “liquid biopsy,” “tumor microenvironment,” “antibody-drug conjugates”, “circulating tumor DNA,” and “artificial intelligence”. Only English-language articles were considered. After screening titles, abstracts, and full texts according to predefined inclusion and exclusion criteria—focusing on relevance, methodological quality, and availability of complete data—a total of 43 articles were selected for analysis. Studies not meeting these criteria, including those lacking primary data or addressing non-biomarker endpoints, were excluded. Selected studies included those reporting emerging biomarkers that could be used for diagnosis, prognosis, or therapeutic response prediction of bladder cancer. The included articles were reviewed, and key findings were extracted, compared, and synthesized to provide an integrated overview of emerging biomarkers and their potential clinical applicability in bladder cancer. Priority was given to clinical trials, meta-analyses, systematic reviews, and translational research studies. Additionally, hand searching of the reference lists of included studies and relevant reviews was performed to identify other eligible publications.

Data extraction focused on the type of biomarkers, mechanisms of detection assay, and tools used to detect them, study population, clinical endpoints, methods for statistics, and validation strategy.

The search was performed by F. A.-A and R. S.-P. B. Any discrepancies among the reviewers were solved by consensus with B. H.-I. and F. G.-R.

## 3. Synthesis of the Evidence

Molecular taxonomy, immune biomarkers, liquid biopsy, and tumor microenvironment (TME) profiling have emerged as complementary pillars in the precision oncology of bladder cancer. Taxonomic classification based on multi-omics analyses enables refined molecular subtyping, improving prognostic accuracy and therapeutic stratification. Immune biomarkers, including programmed death 1 (PD-1) and its ligand (PD-L1), tumor mutational burden (TMB), and novel checkpoint targets, inform patient selection for immunotherapy and combination strategies. Liquid biopsy approaches, particularly circulating tumor DNA and exosomal profiling, allow non-invasive, real-time monitoring of tumor evolution and treatment response. Finally, TME characterization reveals the dynamic interplay between cancer cells and immune-suppressive populations, offering novel targets to overcome therapeutic resistance.

### 3.1. Molecular Subtyping (Taxonomic Groups)

**Figure 1:**
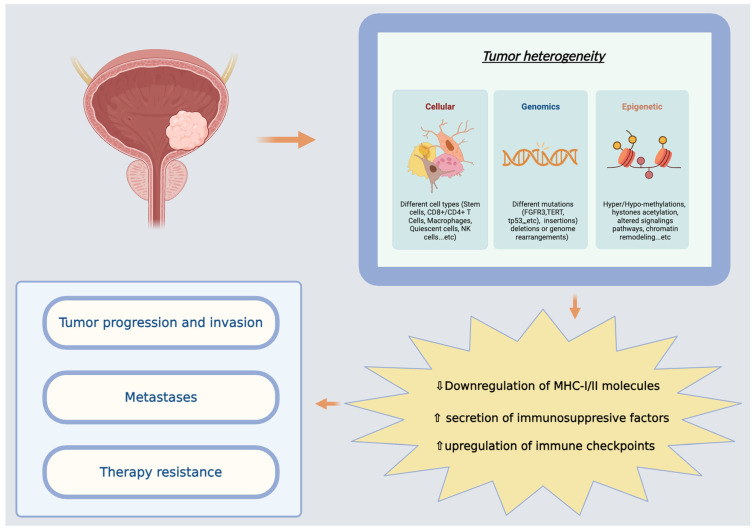
Schematic representation of tumor heterogeneity in bladder cancer. Cellular, genomic, and epigenetic diversity drives tumor progression, metastasis, therapeutic resistance, and immune evasion, ultimately influencing clinical outcomes and therapeutic decision-making.

Our understanding of the heterogeneity of BC has rapidly advanced following the application of molecular taxonomic methods (Figure 1). Molecular taxonomy studies have shown that the major intrinsic subtypes of muscle-invasive bladder cancer—notably the luminal and basal/squamous classes—are primarily distinguished by gene-expression patterns, luminal tumors typically over-express differentiation markers such as GATA3, FOXA1 and uroplakins, and frequently harbor FGFR3 mutations, whereas basal/squamous tumors exhibit high expression of KRT5/6, KRT14, CD44, and commonly display TP53 or RB1 loss [8]. Epigenetic profiling further supports these distinctions, with genome-wide DNA-methylation signatures and subtype-specific histone-modification patterns contributing to enhancer/promoter activity that reinforces luminal versus basal phenotypes [9]. In parallel, cellular heterogeneity also plays a defining role: basal tumors show greater immune and stromal infiltration, whereas luminal tumors tend to be more differentiated with distinct immune-evasion patterns, linking the cellular context of the tumor microenvironment to molecular subtype identity [10]. While early transcriptomic studies proposed basal, luminal, and p53-like subtypes [11], the Cancer Genome Atlas (TCGA) program, based on integrated multi-omics, identified five muscle-invasive bladder cancer (MIBC) molecular classes: luminal, luminal-papillary, luminal-infiltrated, basal/squamous, and neuronal [8]. The latter further delineated six classes: luminal papillary (LumP), luminal-nonspecified (LumNS), luminal-unstable (LumU), stroma-rich (SR), basal/squamous (Ba/Sq), and neuroendocrine-like (NE-like); they are based on associations with oncogenic drivers, immune/stromal infiltrations, histopathological traits, and clinical outcomes [12]. Findings from similar integrative analyses have delineated prognostic molecular characteristics for molecular subtypes of NMIBC [13]. Originally inferred from transcriptomic analyses, molecular classification can now be computationally approximated by traditional immunohistochemistry (IHC) on routine pathology specimens. IHC testing for basal (CD44, CK5/6 markers), luminal (GATA3, CK20 markers), and neuroendocrine-like subtypes (Synaptpophysin, chromogranin A, CD56 markers), which are associated with specific clinical behavior, prognosis, and potential therapeutic implications, is thus both a practical and feasible way to assess molecular subtype in archival tumors [14]. This surrogate classification allows for the incorporation of molecular taxonomy in daily clinical routine practice, enabling a more accurate risk stratification and personalized treatment choices. As a result, molecular classifications were proposed to assign tumors into clinically and biologically distinct groups associated with prognosis, as well as response to platinum-based chemotherapy or immune checkpoint inhibitors (ICIs).

Basal bladder cancer patients are associated with aggressive tumor behavior, high-grade, and poor overall survival (OS), whereas luminal bladder cancer patients have better outcomes. In most of the luminal subtypes, an “intensive” tumor-infiltrating lymphocytes (TILs) pattern was not observed, while in the case of the basal subtype, its prevalence was higher. To assess this, the authors also studied the association of the TIL patterns according to molecular subtypes with OS [15]. They observed that by comparing basal subtypes that lacked intensive TILs to basal subtypes with intensive TILs, the prognosis was worse for the basal subtype without intensive TILs. When the TIL intensity was non-intensive, OS was longer for the luminal subtype. High TILs were significantly associated with improved survival, especially in basal-like subtype tumors. On the other hand, luminal-infiltrated bladder cancer has been associated with poor prognosis, regardless of whether neoadjuvant chemotherapy (NAC) was given. This emphasizes the differential effect of TILs in various molecular subtypes of bladder cancer and their potential function as a prognostic factor. Despite demonstrating diagnostic and prognostic value, the evidence is weakened by methodological variability and insufficient replication across independent populations.

In other work, TCGA exome data was used to identify class-specific mutations and the set of available copy number profiles, organized by consensus class, to identify class-specific copy number aberrations (CNAs) [16]. They composed all CNAs, gene fusion, and gene mutation to complete the genomic alteration profile for each of the bladder cancer genes (Table 1).

LumP tumors were predominantly FGFR3 mutation-enriched. Combining mutation, fusion, and copy number amplification events, FGFR3 genomic alterations were found to be enriched in over 55% LumP tumors. LumP tumors also exhibited significantly more common KDM6A mutations than other subtypes (>30% of cases). Multiplex ligation-dependent probe amplification (MLPA) status of cyclin-dependent kinase inhibitor 2A (CDKN2A) and CNA data for identified homozygous/deep deletions (HDs) of CDKN2A in 33% of LumP tumors, which was significantly higher than in any other class.

The LumNS class was substantially distinguished by mutations in EE74, such as ETS Transcription Factor 3 (ELF3), an early regulator of urothelial differentiation activated by Peroxisome Proliferator-Activated Receptor Gamma (PPARG) [15]. There was also a significant alteration of PPARG, with over 2/3 of LumNS tumors having amplifications or fusions.

LumU tumors also demonstrated a high frequency of PPARG mutations, even more than in LumNS (89% vs. 76%). In addition, Erb-B2 receptor tyrosine kinase 2 (ERBB2) amplification was common in LumU tumors, and its mutation did not harbor a significant association with any of the consensus classes. In contrast to other luminal classes, LumU tumors exhibited TP53 and excision repair cross-complementation group 2 (ERCC2) mutations in most cases. More broadly, LumU was the group with the most genomic alterations, showing the highest frequency of somatic mutation.

For Ba/Sq tumors, as suggested by the existing literature, the most common mutations identified from TCGA exome data were TP53 and RB1 [17]. Ba/Sq tumors showed markedly elevated expression of epidermal growth factor receptor (EGFR) and its ligands, consistent with a potential sensitivity to EGFR-targeted therapies, as previously demonstrated in both in vitro and in vivo studies. In addition, Ba/Sq tumors exhibited strong expression of immune checkpoint markers and antigen-presenting machinery genes, suggesting an increased likelihood of response to immunotherapies. Conversely, NE-like and LumU tumors displayed profiles indicative of potential radiosensitivity [18,19].

In other consensus class-based retrospective analysis of patient outcomes after NAC and treatment with the anti–PD-L1 antibody atezolizumab (IMvigor210), outcomes correlated with consensus class in NAC-free patients, but no significant associations were observed among NAC-treated patients [20]. Nonetheless, comparison of survival curves suggested that individuals with Ba/Sq or LumNS tumors may derive greater benefit from NAC, while those with SR tumors may not. Furthermore, atezolizumab responders were enriched among patients with LumNS, LumU, and NE-like tumors, with recent evidence highlighting NE-like tumors as particularly susceptible to immune checkpoint blockade.

Trials in progress are currently prospectively validating the ability of these subtypes to select patients for treatment and predict benefit.

### 3.2. Pathway-Specific Molecular Alterations

The pathogenesis of bladder cancer is influenced by alterations occurring in specific molecular pathways that lead to uncontrolled cellular division. Such molecular alterations may lend themselves to therapeutic interventions (Figure 1).

#### 3.2.1. Cell Signaling and Gene Regulation

Cell cycle dysregulation is identified at the central hallmark level pathways. One of them is TP53 (located on chromosome 17p13.1), which effectively inhibits cell cycle progression through transcriptional activation of p21WAF1/CIP1 [13]. Although mutations and functional inactivation of this gene are present in up to 76% of MIBC, its adverse prognostic significance remains controversial.

Wild-type p53 has a short half-life that precludes its accumulation in the cell nucleus. Instead, mutant p53 is resistant to degradation and hence accumulates at higher levels in the nuclei, which can be identified by immunohistochemical staining [21,22]. Plus, loss of p21 (cyclin-dependent kinase) expression, amplification of mouse double-minute two homolog (MDM2), and inactivation of RB are mediated with constant proliferation [23]. Decreased caspase-3 activity, overexpression of surviving, and imbalances in the Bcl-2 family have been involved in the resistance to apoptosis, anti-apoptotic Bcl-2 portend poor results, while pro-apoptotic Bax is associated with improved survival [24,25].

In terms of cell signaling and gene regulation, aberrant activation of signaling pathways (notably FGFR3 mutations or fusions, HRAS overexpression, and alterations in MAPK components such as MAP4K3) further promotes tumor growth and progression [26]. A multi-gene analysis of molecular pathways (69 genes involved in known cancer pathways) also proved that overexpression of JUN, MAP2K6, STAT3, or ICAM1 in BC patients, 5-year OS was clearly defeated if more than one gene was overexpressed, in contrast with low/normal expression in three or more (5% vs. 61%) [27,28].

Other studies have hypothesized about sex hormone receptors as prognostic factors, showing that lower levels of estrogen receptor-beta expression have been associated with better progression-free survival rates in NMIBC disease, while higher levels have been associated with advanced tumors [29].

Nowadays, other transducer signaling studied, such as trophoblast cell-surface antigen 2 (TROP-2), have demonstrated their implication in tumor aggressiveness in BC [30]. Nectin-4 is a transmembrane protein involved in making and maintaining adherens junctions, although it is not expressed in high levels in adult somatic cells, it is upregulated in bladder cancer. Whereas previous studies had mainly concentrated on primary UC and its histological variants, a recent study provides new insights into Nectin-4 and TROP-2 expression in more aggressive metastatic forms for which therapeutic options were essentially lacking. TROP-2 was found to be a frequently expressed target (>90%) in both the membrane and cytoplasm of UC with squamous differentiation, and it was also robustly detected in plasmacytoid UC. A recent study found higher levels of TROP-2 in most BC subtypes (except in the neuroendocrine subtype) and revealed that after prolonged Enfortumab Vedotin (EV) exposure, cells can downregulate Nectin-4, leading to EV resistance, but retain TROP-2 expression and remain sensitive to sacituzumab govitecan, suggesting non-overlapping resistance mechanisms to ADCs [31,32].

#### 3.2.2. Inflammation, Angiogenesis, and Immune Modulation

In addition, inflammation and immune modulation are also employed by BC to promote progression. Elevated interleukin-6 levels (IL-6) and increased C-reactive protein (CRP) are related to more advanced stages and worse survival [33,34]. The study carried out by Andrews B. et al. showed that IL-6 and IL-6 soluble receptor (IL6sR), which potentiates systemic effects of IL-6, were correlated with unfavorable pathological characteristics, such as muscle invasion, lymph vascular infiltration, and lymph node metastasis. Certainly, in their multivariate analyses, both IL-6 and IL-6sR emerged as independent predictors of lymph vascular invasion, lymph node metastasis, disease recurrence, and disease-specific survival [35]. One of the most important markers is PD-1/PD-L1. Its expression in tumor and immune cells allows the tumor to escape immune editing and is targeted by immune checkpoint blockade with the PD-1/PD-L1 inhibitors in advanced disease [36,37].

Angiogenesis, predominantly driven by the overexpression of VEGF in over 80% of tumors, correlates with recurrence, progression, and adverse pathologic features. Specifically, the value of concurrent inhibition of pro-angiogenic factor placental growth factor (PGF) and VEGF-A significantly enhances the efficacy of immunotherapy in bladder cancer. This combined strategy reduces tumor angiogenesis and increases CD8^+^ cytotoxic T cell infiltration, offering a promising therapeutic synergy [38]. Deepening in this field, authors showed that angiogenesis can also be quantified by microvessel density. Some studies demonstrated that microvessel density was associated with disease progression in patients with organ-confined tumors, tumors extending through the bladder wall, and tumors that had spread to regional lymph nodes, serving as an independent prognostic factor for OS [39,40].

The ability of bladder cancer cells to invade lymphatic and blood vessels determines how well they will be able to metastasize. Invasion is facilitated by proteins involved in epithelial cell–cell adhesion, of which E-cadherin is a key member. Loss of E-cadherin, overexpression of MMP-2 and MMP-9, and upregulation of adhesion molecules such as ICAM1 and Nectin-4, the latter with special relevance nowadays, as it serves as a therapeutic target for ADCs such as EV [41,42,43].

Elevations in carbohydrate antigen 19-9 (CA 19-9) and carcinoembryonic antigen (CEA) also associate with higher recurrence rates and poorer survival. The first study about tumor markers related to response to NAC showed that patients with persistently elevated markers following NAC have a very poor prognosis following cystectomy, which may help identify chemotherapy-resistant tumors. In addition, responder patients after cystectomy with normal CEA and CA 19.9 had an overall and recurrence-free survival better than non-responder ones [44]. These biomarkers show promising results in early studies; however, the absence of standardized analytical protocols hampers reproducibility.

#### 3.2.3. Immune Profile and Targeted Therapies

Immune profiling is increasingly recognized as a complementary component of a personalized approach in UC. Numerous biomarkers provide information on the tumor microenvironment and its capacity to respond to immunomodulatory therapies [45]. These immunological parameters are often closely aligned with molecular subtypes, reinforcing the value of combined profiling strategies to guide individualized therapy.

Over the last years, the therapeutic landscape in urothelial bladder cancer has drastically changed, with ICIs and ADCs becoming a part of our daily practice, marking a paradigm shift toward precision-guided systemic therapies.

ICIs, especially PD-1/PD-L1 directed agents, provide clinical benefit in UC. Agents such as pembrolizumab (anti-PD-1) and atezolizumab (anti-PD-L1) have been approved as first-line treatments for metastatic MIBC in cisplatin-ineligible patients, and as second-line options for metastatic disease [46,47]. Additionally, avelumab is now used as maintenance therapy following an initial response to platinum-based chemotherapy [48].

More recently, nivolumab (antiPD-L1/2) has been approved for use as an adjuvant therapy after radical cystectomy in high-risk non-metastatic advanced MIBC (ypT2-4, pT3-4 or N+). This approval is supported by the phase III CheckMate 274 trial, which demonstrated significantly improved disease-free survival compared to placebo, particularly in patients with PD-L1 expression ≥ 1% [49].

Beyond the PD/PDL-1 marker, specific molecular alterations can also be managed with targeted therapies. The oral pan-FGFR tyrosine kinase inhibitor erdafitinib was approved for patients with locally advanced or metastatic bladder cancer harboring FGFR2 or FGFR3 mutations or fusions, based on the results of the THOR trial. This study showed an objective response rate of 40%, highlighting the clinical utility of molecular profiling to identify actionable alterations [50].

More recently, ADCs have emerged as promising agents in the treatment of advanced UC. In the Phase III EV-302 trial, the combination of EV with pembrolizumab significantly improved OS and progression-free survival compared to platinum-based chemotherapy in previously untreated cisplatin-ineligible patients with locally advanced or metastatic disease [51]. This combination has become a new standard first-line therapy for selected patients, and it has transformed the treatment paradigm for metastatic urothelial cancer, setting a new benchmark and driving more trials testing combination strategies.

Nowadays, given the differences in ADCs (and combinations) mechanisms of action, outcomes are not the same. New and promising ADCs therapies such as Sactizumab Govitecan (humanized monoclonal antibody targeting TROP-2 conjugated to SN-38 drug, the active metabolite of irinotecan), Trastuzumab Deruxtecan, or Disitamab Vedotin (Human Epidermal growth factor Receptor 2 [HER2]) ADCs are being tested in advanced/metastatic urothelial (mUC) cancer trials [52,53,54]. Sacituzumab Govitecan was the first to receive accelerated FDA approval for mUC with prior platinum and IO pretreatment; however, the indication was withdrawn last year. Numerous efforts are carried out to improve global access, optimize patient selection, and choose the correct treatment sequencing.

### 3.3. Liquid Biopsy

LB has recently emerged as an innovative approach in precision oncology for detecting tumor-derived components in body fluids such as blood and urine [55]. LB technologies, whose ultra-high sensitivity can provide wide proteomic profiling, in-depth quantification of RNA species (e.g., circulating tumor cells [CTCs], cell-free DNA [cfDNA], RNA species [microRNAs, long non-coding RNAs], extracellular vesicles, and tumor-associated proteins). This test allows valuable insights into tumor dynamics without the need for repeated invasive procedures [56,57,58,59,60].

The current gold standard of diagnosis (cystoscopy and biopsy) is invasive, expensive, and carries patient anxiety and procedural risks [61]. Given these limitations and the frequent need for lifelong monitoring, there is an unmet need for non-invasive, reliable tools for early diagnosis, dynamic follow-up, and treatment guidance.

In BC, this is especially relevant since the urothelial cells are in direct contact with urine. LB allows real-time tumor monitoring, detection of minimal residual disease, prediction of recurrence, and assessment of treatment response [62]. In last years, advances in high-throughput technologies have enabled the identification of urine-based metabolomic and proteomic biomarkers with markedly improved diagnostic accuracy. When combined with artificial intelligence algorithms, these molecular profiles allow for more precise risk stratification and non-invasive monitoring of bladder cancer patients. Moreover, integrative multi-omics approaches (including transcriptomics, DNA methylation, and proteomics) have demonstrated substantial potential for detecting minimal residual disease and predicting recurrence earlier than conventional methods [63,64,65].

Genomic analyses have identified recurrent mutations such as FGFR3 and TERT promoter alterations, as well as DNA methylation signatures, associated with disease subtype and prognosis [66,67]. Transcriptomic profiling has revealed diagnostic and prognostic microRNA panels, while proteomic studies highlight markers like NMP22 and surviving [67,68]. Extracellular vesicles analysis has expanded the biomarker repertoire, providing insights into tumor biology and potential therapeutic targets [69].

**Figure 2:**
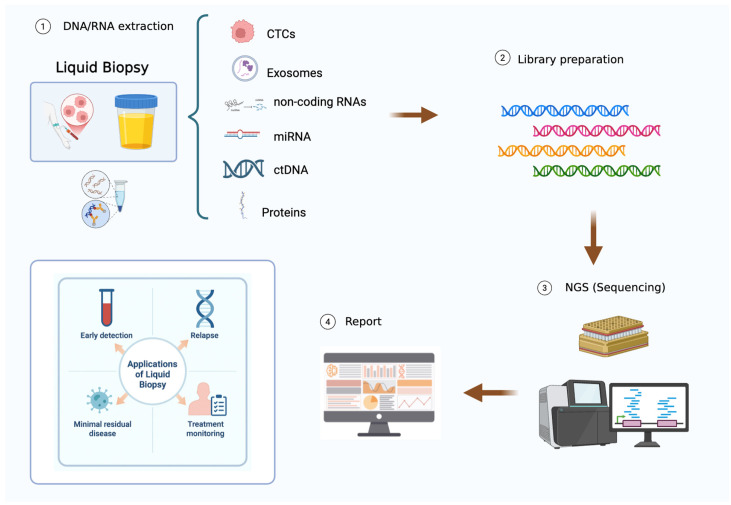
Applicability of LB in DNA sequencing and clinical outcomes.

In addition, the diagnostic and predictive accuracy of LB has been improved by multi-omics integration and advanced computational approaches, including machine learning beyond single-marker detection [70,71]. These strategies enable the identification of actionable targets, guiding precision oncology in BC by tailoring therapies to individual tumor profiles (Figure 2). LB provides an interesting new opportunity for longitudinal assessment, including the monitoring of clonal evolution and mechanisms of resistance, without the need for repeated invasive procedures [55].

Clinical applications of LB in BC include several contexts, such as early diagnosis, prognosis, therapy guidance, or treatment monitoring. However, several challenges remain before LB becomes a routine clinical tool: pre-analytical variability in sample collection and processing, assay standardization, reproducibility, and the need for prospective validation in large and diverse cohorts [72].

Six urinary diagnostic assays for BC have been approved for clinical use by the FDA: qualitative bladder tumor antigen (BTA) (BTA stat), quantitative BTA (BTA TRAK), quantitative nuclear matrix protein 22 (NMP22) (Alere NMP22), qualitative NMP22 (BladderCheck), fluorescent immunohistochemistry (ImmunoCyt), and fluorescence in situ hybridization (FISH) (UroVysion) [72,73,74,75]. Of these, three identify genetic or protein changes in exfoliated urinary cells (UroVysion, BladderCheck, and ImmunoCyt), and two measure marker proteins secreted into urine (NMP22 and BTA assays). In the past decade, in addition to the FDA-approved kits described above, numerous other tests have been developed for the study and investigation of biomarkers in urine [66,76,77,78,79,80,81,82,83,84,85,86,87,88,89,90,91].

The methods used for detection and surveillance of bladder cancer work based on DNA mutations or methylation, RNA signatures, improved cytology, and protein-based assays (Table 2).

Deepening in Bladder Epicheck, its software analyzes the data obtained by qPCR. As noted by Leonardi et al., EpiScore, with values ranging from 0 to 100, allows for the prediction of the outcome. Episcore determination < 60 provides a very high negative predictive value for tumor recurrence, whilst Episcore ≥ 60 indicates a positive result, leading to additional interventions (such as cystoscopy) to rule out bladder cancer recurrence [92]. Although the current landscape of biomarkers in bladder cancer demonstrates significant promise, it remains hampered by limited prospective validation and real-world clinical deployment.

The successful translation of biomarkers from the laboratory to clinical practice requires robust validation in multicenter settings. Recent trials have shown that molecular urine tests can achieve sensitivities and specificities comparable to, or exceeding, those of cystoscopy in the surveillance of NMIBC. In addition, even more heterogeneous AI- and machine learning-based models have been validated in diverse, multicenter cohorts, further supporting their practicality and generalizability, which adds further support for such tools to be included in clinical practice guidelines [93].

Recent advances in molecular biology and bioinformatics are accelerating their integration into clinical practice, with the potential to shift BC care towards a truly personalized approach [13,94]. Next-generation biomarker validation, standardization of analytical assays, and evidence of clinical utility in randomized prospective trials should drive the next investigations.

#### New Perspectives About ctDNA

Ongoing clinical trials are exploring the integration of molecular subtyping and ctDNA to optimize personalized therapeutic strategies in BC. These trials are evaluating the utility of liquid biopsy to detect MRD after radical treatment or in bladder-sparing treatments. In addition, by using tumor-informed ctDNA assays (PCR or NGS panels) to stratify MIBC patients, clinical trials propose to select patients likely to benefit from targeted therapies (Table 3) [47,95,96,97,98,99,100,101,102]. Because MIBC is increasing its incidence and due to its high mortality and recurrence rate, several trials in the past years have been developed with the aim of improving oncologic results in this field, focusing on the role of ctDNA as a predictive and prognostic biomarker.

Adaptive clinical trial designs seek to determine whether tailoring ICIs can eradicate detectable ctDNA, thereby improving survival and, in selected cases, achieving cure. Equally necessary are prospective trials aimed at validating ctDNA as a reliable surrogate endpoint, both for prognosis and for predicting therapeutic response in MRD settings. These studies would be very useful with respect to adapting treatment, especially now with the inclusion of newer.

In the future, an integrative approach of ctDNA analysis with other molecular layers, including genomic, epigenomic, and other multi-omics signatures, will increase sensitivity and specificity for MRD identification. However, this raises a relevant clinical challenge: what is the best approach for patients who have a positive or rising ctDNA in the absence of visible metastases on cross-sectional imaging? This underscores the need for robust, biomarker-driven interventional studies, which, as stated previously, are underway.

As novelty, BISCAY used a biomarker-directed platform trial design with combinations of durvalumab and serial ctDNA surveillance. Progression-free survival was similar across treatment arms, but molecular tracking of ctDNA levels and FGFR mutations revealed associations with clinical outcomes [103]. Beyond blood-based assays, urine-derived tumor DNA is emerging as a complementary, non-invasive modality for disease monitoring [104]. Despite encouraging preliminary data, the biomarker’s predictive value remains uncertain until validated using larger, prospective, real-world datasets

### 3.4. Tumor Microenvironment (TME)

Deepening in the biomolecular setting, recent advances in cancer biology and immunology have significantly improved our understanding of the TME and its impact on the host immune response. The dynamic interplay between tumor cells and their surrounding stroma is now recognized as a critical determinant of cancer progression, metastasis, immune evasion, and response to immunotherapy. Recent research increasingly focuses on immunosuppressive characteristics of the TME, particularly regarding patterns of immune cell infiltration and the regulation of immune checkpoints [45,105]. Tumors seem to create an immunologically unfavorable microenvironment that suppresses antitumor immunity by deploying many different mechanisms, which inhibit the formation of potent immune responses.

Recent evidence underscores the pivotal role of immunological biomarkers in predicting and monitoring response to ICIs in urothelial carcinoma. PD-L1 expression, assessed by immunohistochemistry in both tumor and immune cells, remains the most established predictive marker, although its prognostic value is influenced by tumor heterogeneity and dynamic modulation during treatment.

High densities of CD8+ TILs and favorable CD8+/FOXP3+ Treg ratios have been associated with improved outcomes following PD-1/PD-L1 blockade, while increased myeloid-derived suppressor cells (MDSCs) and M2-polarized macrophages correlate with resistance [106,107]. TMB and neoantigen load, particularly when integrated with molecular subtype classification, provide additional predictive accuracy, with basal/squamous subtypes generally exhibiting greater immunogenicity and responsiveness to ICIs [108].

Emerging data from prospective trials, including IMvigor211, CheckMate 274, and JAVELIN Bladder 100, confirm that combining PD-L1 expression with composite immune scores or TMB enhances patient selection beyond single-marker strategies. Moreover, integration of longitudinal ctDNA dynamics with immunological profiling is emerging as a powerful tool for early detection of ICI resistance and disease progression. Together, these findings highlight the potential need to include immune biomarker assessment into standard clinical practice to optimize the therapeutic benefit of ICIs and to facilitate the development of personalized, biology-driven immuno-oncology approaches in bladder cancer. In addition, a better characterization of the cell composition and heterogeneity of the bladder cancer TME should improve patient stratification.

Thus, strategies aimed at remodeling the TME and restoring effective immune function are key to optimizing the success of cancer immunotherapies. A comprehensive understanding of the pathways underlying immune escape and resistance and the identification of novel molecular targets capable of counteracting tumor-driven immune tolerance could open new therapeutic approaches, thereby improving clinical prognosis.

## 4. Future Directions

The future of urothelial carcinoma management will be marked by molecular biomarkers, AI analytics, and rigorous multicenter real-world validation in the clinical setting. High-throughput multi-omics approaches (from genomics, transcriptomics, to proteomics and metabolomics) should be standardized across platforms to provide reproducible and comparable results. AI-based predictive models, especially when combined with radiomics, digital pathology, and longitudinal liquid biopsy data, bring the potential to improve patient stratification and treatment selection; however, broad availability will be based on transparent algorithms, interpretability, and approval by regulatory bodies.

### 4.1. Emerging-Directed Therapies

Today, emerging immune checkpoints outside of the PD-1/PD-L1 axis, including lymphocyte-activation gene 3 (LAG-3), T cell immunoreceptor with Ig and ITIM domains (TIGIT), and B7 homolog 3 (B7-H3), are increasing the interest in UC management. LAG-3 is a T cell inhibitory receptor that downregulates T cell proliferation and cytokine production, frequently co-expressed with PD-1 in exhausted T cells, suggesting potential synergistic blockade strategies [109]. TIGIT is an inhibitory receptor expressed on T cells and natural killer cells and regulates anti-tumor immunity through competition with CD226 for binding to CD155, and increased expression of TIGIT in bladder tumors is associated with impaired effector function. As a B7 family member, B7-H3 performs an immunosuppressive function in the TME, and poor prognostic features and increased metastasis associated with B7-H3 have been reported [110,111,112]. Preclinical models suggest that inhibition of these pathways, used in conjunction with or independent of PD-1/PD-L1 inhibitors, may further activate the immune response and bypass subsequent resistance to ICIs.

Several early-phase clinical trials are evaluating monoclonal and bispecific antibodies against these targets as next-generation immuno-oncology agents in bladder cancer [113]. These markers hold promise for refining ICI selection and guiding rational combination strategies. Current evidence suggests potential utility, but further multi-center studies are essential to substantiate its performance in diverse patient populations.

### 4.2. AI Application in BC

AI has gained increasing interest as a disruptive tool with the potential to radically change diagnostics, treatment targeting, patient supervision, and may also be used for administrative work. AI applications in healthcare are already making significant strides in fields such as radiology, oncology, and pathology [114].

Recent advances in AI have opened new pathways for integrating complex biomarker datasets in bladder cancer. Specifically, AI-based models can process high-dimensional multi-omic data—including gene expression signatures linked to molecular taxonomies (e.g., basal vs. luminal subtypes), immune-infiltration profiles, and liquid-biopsy derived analytes such as circulating tumor DNA (ctDNA), miRNAs, or exosomes. By combining the molecular classification of tumor subtypes, immune landscapes, and minimally invasive read-outs from liquid biopsies, AI frameworks offer the potential to refine risk-stratification, predict therapeutic response, and monitor disease evolution in bladder cancer [115]. For example, after defining tumor taxonomy and immune status, a liquid-biopsy panel of candidate biomarkers may be fed into machine-learning or deep-learning algorithms to generate predictive risk scores or decision-support tools, thereby bridging the gap between molecular biology and clinical application [116]. In this way, AI acts as the connective tissue between biochemical taxonomy, immune microenvironment, and biomarker measurement, enabling a truly integrated precision oncology approach for bladder cancer.

AI is increasingly changing the diagnosis and treatment of bladder cancer, but some critical difficulties need to be addressed to promote its applicability to clinical work. A new study in a diagnostic setting, Cystoscopy AI Diagnostic System (CAIDS), a stable AI model-based application, has been trained on over 69,000 images of 10,729 patients and showed an accuracy of 93.9% and sensitivity of 95.4%, surpassing experienced urologists in real-time detection of a lesion [117]. Likewise, convolutional neural networks (CNN)-based models such as CystoNet achieved per-frame sensitivities of 90.9% and specificities of 98.6%, facilitating superior intraoperative navigation and tumor identification. These models evaluate the characteristics of bladder tissue samples, such as cell morphology and tumor grade, to assess the likelihood of recurrence after treatment [118]. Next steps are the implementation of prospective multicenter validation of such systems and incorporation into standard cystoscopy systems, which might reduce rates of recurrence owing to an increased resection completeness.

In CT imaging, AI demonstrates very good predictive potential for staging. Deep learning models using CT images yielded good preoperative discriminative performance of MIBC vs. NMIBC, and multi-task learning models based on MRI showed higher reliability in evaluating muscle infiltration than single-task models. Radiogenomics-integrated MRI and RNA-seq-only data achieved the best staging performance (92% accuracy, 94% sensitivity, and 88% specificity), with a gain of 17–33% as compared with models trained on single-modality data [119]. These results highlight the necessity of multimodal AI pipelines that integrate imaging, molecular, and clinical information for precision staging and therapeutic response assessment.

One of the most clinically relevant potential applications could be in urine-based diagnostics. With a sensitivity ranging from 92.2 to 100% in retrospective cohorts and of 89.6% in a prospective cohort, the PUCAS (Precision Urine Cytology AI Solution) technology led to a decreased frequency of cystoscopy by 57.5% with a negative predictive value of 96.4% [120]. Correspondingly, 93% for high-grade tumors and 66.7% for low-grade disease sensitivity were obtained by the VisioCyt assay, demonstrating the current inability to detect indolent lesions [121]. Supervised machine learning predictors built on >1000 NMIBC patients receiving Bacillus Calmette–Guérin (BCG) have recently shown enhanced prediction of recurrence and progression with respect to Sylvester Risk Tables [118]. This highlights the possibility for AI not only to stratify oncologic risk but also to tailor personalized therapy for non-malignant bladder diseases.

And from another point of view, in the paper by Mahapatra et al., it is pointed out that AI as applied to bladder cancer should be incorporated into a broader picture of the bladder pathophysiology, such as overactive bladder, neurogenic bladder, and bladder outlet obstruction [122].

Moving forward, three strategic priorities become evident: (1) multicenter validation and data sharing to combat heterogeneity and small sampling biases; (2) explainability and interpretability to undercut the “black box” challenge and to encourage clinical trust; and (3) integration within precision oncology via radiogenomics, digital pathology, and molecular biomarker-driven AI pipelines. These methods may expedite the migration of AI tools from the experimental realm to standard-of-care, thereby improving diagnostic performance, treatment individualization, and prognosis of bladder cancer patients. Future investigation should focus on the algorithm stability of tumor identification, harmonization of data across centers, and regulatory pathways for in vitro diagnostic implementation.

Cost-effectiveness analyses and health technology assessments will be critical to justify the incorporation of novel diagnostics into public health systems. Finally, collaborative networks linking academic centers, industry, and regulatory agencies will be essential to accelerate translation from discovery to implementation, enabling a new era of precision oncology in bladder cancer.

## 5. Conclusions

The changing paradigm of bladder cancer treatment is driven by progress in molecular subtypes, immune biomarkers, liquid biopsy, and artificial intelligence. Together, these advancements provide new opportunities for early detection, better assessment of risk, and individualized therapeutic approaches.

Combining multi-omics data with computational analysis and minimally invasive approaches can help optimize precise and personalized clinical management that is adapted to tumor heterogeneity. However, translating these advances into routine practice will require multicenter validation, methodological standardization, and cost-effectiveness analyses to ensure broad applicability and equity of access.

Ultimately, the convergence of biology-driven discoveries and computational advancement will drive to a new world of precision oncology that has strong potential to impact outcomes for patients with UC.

## Figures and Tables

**Table 1:** Genomic alterations, clinical associations, and therapeutic implications across consensus molecular subtypes of muscle-invasive bladder cancer.

Molecular Subtype	Key Biomarkers	Clinical Implications	Therapeutic/Response Implications
LumP	FGFR3 mutations/fusions/amplifications (~55%), KDM6A mutations (~38%), CDKN2A deep deletions (~33%)	24–35% of MIBC. Most favorable prognosis (5-year OS ≈ 65–70%); enriched in younger patients and T2 tumors.	Likely sensitive to FGFR3 inhibitors (erdafitinib, BGJ398); potential biomarker for targeted therapy
LumNS	ELF3 mutations (~35%), PPARG alterations (amplifications/fusions ~76%)	8–12% of MIBC. Older patients (>80 yr), micropapillary histology, carcinoma in situ association. Intermmediate outcomes(5-year OS ≈ 50–55%)	Potential benefit from NAC; some enrichment in atezolizumab responders
LumU	PPARG alterations (~89%), ERBB2 amplification (~39%), TP53 mutations (~76%), ERCC2 mutations (~22%), high CNA and mutation load	15–20% of MIBC. Most genomically altered class; poor prognosis trend; high cell cycle activity. Inferior survival (5-year OS ≈ 40–45%)	Potential sensitivity to ERBB2-targeted therapy; association with radiotherapy response; enrichment for atezolizumab response
Ba/Sq	TP53 mutations (~61%), RB1 mutations (~25%), concurrent TP53+RB1 in ~14%, EGFR pathway activation, STAT3/HIF1A regulon activity	20–35% of MIBC. Enriched in females, advanced stage, squamous differentiation; poor prognosis (5-year OS ≈ 35–45%).	Potential sensitivity to EGFR inhibitors; high immune infiltration and immune checkpoint expression → candidates for immunotherapy; possible benefit from NAC
NE-like	Ubiquitous TP53 (94%) + RB1 (94%) alterations, high cell cycle activity, neuroendocrine histology (~72%)	Worst prognosis (median OS < 24 months even with multimodal therapy), highly aggressive, rare (~3%)	Potential sensitivity to ICIs; potential radiotherapy responders; parallels with small cell carcinoma treatment
SR	High stromal gene expression, no specific driver mutation	~15% of MIBC. Intermediate survival (5-year OS ≈ 50%). Histology is dominated by stroma	Immune infiltration (T/B cell) but lower ICI response; limited sensitivity to NAC

**Table 2:** Summary of urinary biomarkers. Abbreviations—CEA: carcinoembryonic antigen; MAUB: mucin antigen of the urinary bladder; FDA: Food and Drug Administration; FISH: fluorescence in situ hybridization; BCG: Bacillus Calmette–Guérin; CE: Conformitée Europeenne; NGS: next-generation sequencing; BS-Seq: bisulfite sequencing; PCR: polymerase chain reaction; SafeSeqS: Safe-Sequencing System; HTS: high-throughput sequencing; RT: real-time; MASO: multiplex allele-specific, oligonucleotide; qPCR: quantitative polymerase chain reaction; ELISA: enzyme-linked immunosorbent assay; POC: point of care.

Test	Variable	Biomarker	Assay	Clinical Application	Sensibility/Specificity	Reference
Urovysion	Chromosome 3-7-9-17	DNA/Sediment cells	FISH	Post BCG/early recurrence	69%/76%	[73]
Immunocyt	CEA, MAUB	Antigens and sulfated mucin glycoproteins (sediment cells)	Immunofluorescence cytology	LG-NMIBC diagnosis	77.5%/62.5%	[74]
Uromark	Epigenetic alterations	Sediment cells/DNA	NGS + BS-Seq PCR	Predictive and monitoring treatment	95%/96%	[76]
NMP22 (Bladder Chek)	NMP22	Protein	ELISA + POC immunoassay	Early diagnosis and monitoring HG recurrence	59%/93%	[68,75]
Uromonitor	FGFR3, TERT, KRAS	DNA	PCR	Predictive (recurrence)	73.5%/93.2%	[78,91]
Uromutert	TERT	DNA	NGS PCR	Early diagnosis	87.1%/94.7%	[79]
Bladder Epicheck	DNA methylation	DNA	RT-PCR	Early diagnosis of HG-NMIBC	81%/83%	[80]
Uroseek	TERT, FGRF3, TP53, CDKN2A, ERB2, HRAS, PIK3CA, METH, BHL, MLL	DNA	SafeSeqS	Early diagnosis and monitoring response	95%/93%	[66]
Urodiag	FGFR3, HS3ST2, SEPT9, SLIT2	DNA	DNA methylation + MASO-PCR	Monitoring HG recurrence	95.5%/75.9%	[81]
CxBladder	CDK1, MDK, HOXA13, IGFBP5, CXCR2	mRNA	qPCR	Early diagnosis	82%/85%	[82,83]
ADXBLADDER	MCM	Protein	ELISA	Predictive (≳T1 disease)	73%/68.4%	[83]
Xpert BC	ABL1, UPK1B, CRH, ANXA10, IGF2	mRNA	RT-PCR	Exclude recurrence	76%/85%	[84]
CYFRA 21.1	Cytokeratin19	Protein	ELISA	Diagnosis	82%/80%	[85]
AssureMDx	FGFR3, TERT, HRAS, OTX1, ONECUT2, TWIST1	DNA	DNA methylation + PCR	Predictive (HG-NMIBC)	93%/86%	[86]
Oncuria	ANG, APOE, A1AT, CA9, IL8, MMP9, PAI1, SDC1, VEGF	Protein	Immunoassay	Diagnosis and follow-up	85%/81%	[87]
UBC Rapid	Cytokeratin 8 and 18	Protein	POC immunoassay	Predictive (Cis)	70.8%/61.4%	[88]
BTA test	BTA	Protein	Immunochromatography + ELISA	Diagnosis and monitoring response	56%/85.7%	[68,89,90]

**Table 3:** Ongoing and published clinical trials evaluating ctDNA as a biomarker in bladder cancer.

Trial	Setting	Drug	Primary Objective	Secondary Objectives	Design	ctDNA Measurement	Preliminary Results
NABUCCO	Neoadjuvant	Ipilimumab + nivolumab	Safety and feasibility	pCR rate and exploratory studies PD-L1 status, TMB, and immune cell infiltration)	Prospective, phase Ib	Before and after neoadjuvant immunotherapy	pCR ~46%; ctDNA clearance correlated with response; ctDNA persistence predicted relapse [95]
ABACUS	Neoadjuvant	Atezolizumab	pCR rate	Disease-free survival (DFS), OS, radiological response (RR)	Prospective, phase II	Baseline and post-neoadjuvant immunotherapy	pCR 31%; ctDNA negativity associated with pCR and better RFS; persistent ctDNA predicted relapse [96]
IMvigor010	Adjuvant	atezolizumab vs. observation	DFS	OS, safety, exploratory biomarker analyses (including ctDNA)	Prospective, phase III, post hoc ctDNA analysis	Post-cystectomy (MRD detection)	No DFS benefit overall; ctDNA+ patients derived benefit from atezolizumab; ctDNA– did not [47]
TOMBOLA	Adjuvant	Atezolizumab	Validation of ctDNA as a biomarker of MRD and predictor of recurrence	Time to recurrence, OS, ctDNA detection vs. radiological imaging	Prospective, observational, multicenter	Baseline, post-surgery, follow-up	ctDNA positivity post-cystectomy predicted early relapse and preceded radiologic recurrence [97]
MODERN	Adjuvant	Nivolumab	DFS in ctDNA-guided adjuvant therapy vs. standard-of-care	OS, cost-effectiveness, safety, quality of life, ctDNA assay validation	Prospective, phase III, randomized	Post-cystectomy	Ongoing [98]
KEYNOTE-361	Advanced/metastatic	Pembrolizumab ± chemotherapy	OS, PFS	Objective response rate (ORR), duration of response (DoR), safety, exploratory biomarkers (including ctDNA)	Prospective, phase III, exploratory biomarker analysis	Baseline and on-treatment	Not meet OS/PFS; ctDNA dynamics associated with survival and treatment response [99]
NCT05979740	Organ-preserving multimodal therapy	Disitamab vedotin + toripalimab + RT	Safety and feasibility	ORR, ctDNA dynamics for recurrence monitoring, recurrence-free survival (RFS)	Prospective, early phase	During treatment and follow-up	Feasible and safe; ctDNA dynamics used to monitor recurrence and treatment response [100]
EV-103	Advanced/metastatic	EV ± pembrolizumab	Safety and efficacy, ORR	Duration of response (DoR), PFS, OS, exploratory biomarkers (ctDNA, PD-L1	Prospective, phase Ib/II	Baseline and during treatment	EV ± pembro: ORR 64.5%, median DoR 13.2 months; exploratory ctDNA analyses ongoing [101]
EV-302/KEYNOTE-A39	Advanced/metastatic	EV + pembrolizumab vs. chemotherapy	OS, PFS	ORR, DoR, safety, quality of life, exploratory biomarkers (including ctDNA)	Prospective, phase III, randomized	Baseline and on-treatment	EV+pembro vs. chemo: OS 31.5 vs. 16.1 months; HR death 0.47; PFS 12.5 vs. 6.3 months; ORR 67.7% vs. 44.4%; ctDNA analysis ongoing [102]

## Data Availability

No new data were created or analyzed in this study. Data sharing is not applicable to this article.
